# Editorial: factors influencing the outcome of total hip and knee arthroplasty

**DOI:** 10.1186/s42836-023-00219-x

**Published:** 2023-12-06

**Authors:** Nicholas D. Clement, Chloe E. H. Scott

**Affiliations:** 1 Edinburgh Orthopaedics, Royal Infirmary of Edinburgh, Little France, Edinburgh, UK; 2 Department of Orthopaedics, University of Edinburgh, Little France, Edinburgh, UK

**Keywords:** Arthroplasty, Factors, Knee, Hip, Outcome

## Abstract

**Background:**

Total hip and knee arthroplasty for end stage arthritis are among the most cost-effective interventions available in all of medicine. Success of hip and knee arthroplasties is not universal and approximately one in ten patients are not satisfied with their arthroplasty and 10 to 34% of patients have an unfavourable long term pain outcome. The aim of this edition of Arthroplasty was to identify factors associated with: (1) poor joint specific outcome, (2) poor health related quality of life outcome and (3) dissatisfaction following total hip and knee arthroplasty.

**Methods:**

The scope was deliberately broad to identify factors (known and unknown) that influence outcome of total hip and knee arthroplasty, and of 40 submissions, eight were selected for this special edition after peer review.

**Results:**

Many of the included studies reported subjective patient reported outcome measures as their key outcomes but other objective measures such as muscle mass, timed Up-and-Go test, kneeling ability and postoperative pain are also featured. Some studies involved a focus on early rehabilitation after surgery (ERAS) principles and how to optimise pre-rehabilitation and reduce length of hospital stay readmission and reoperation. The effect of metal allergy and drain usage on outcomes is also explored. A variety of methodologies have been used including one randomised control trial, some machine learning and three systematic reviews investigating the effect of metal allergy on outcomes, associations with postoperative pain and the effect of patella resurfacing.

**Conclusion:**

This special edition has advanced our knowledge of factors influencing to the outcome of hip and knee arthroplasty but has also identified several areas of research that need to be addressed to improve the outcomes of our patients.

## Introduction

Total hip and knee arthroplasty for end stage arthritis are among the most cost-effective interventions available in all of medicine [1]. The “success” of hip and knee arthroplasty is typically quantified using subjective patient reported outcome measures (PROMs). Other objective measures of outcome such as energy consumption, gait and pace are increasingly being used to measure outcomes in high functioning patients to limit the ceiling effects inherent in many PROMs used in orthopaedics. Quantitative measures are however essential in objectively capturing the patient experience but also in evaluating and investigating new procedures and techniques. PROMs capture the patient’s own evaluation of the outcome of their surgery in a single quantifiable score, which is important as patient and surgeon perceptions of “success” following hip (THA) and knee (KA) arthroplasty are not necessarily aligned. Success of hip and knee arthroplasties is not universal and approximately one in ten patients are not satisfied with their arthroplasty [2] and 10% to 34% of patients have an unfavourable long term pain outcome [3]. It is important to identify which patients are at risk of a poor outcome, not only to inform patients preoperatively as part of their consent process, but to also potentially address these factors in the future to improve their outcome [4]: we need to understand our “failures” if we are to improve.

The aim of this edition of *Arthroplasty* was to identify factors associated with: (1) poor joint specific outcome, (2) poor health related quality of life outcome and (3) dissatisfaction following THA and KA. The scope was deliberately broad to identify factors (known and unknown) that influence outcome of THA and KA, and of 40 submissions, eight were selected for this special edition after peer review (Available on https://www.biomedcentral.com/collections/factors-hka, Accessed date: 10 Oct 2023) [5–12]. Many of the included studies reported PROMs as their key outcomes [7–10] but other objective measures such as muscle mass [5], timed Up-and-Go test [5], kneeling ability [12] and postoperative pain [10] are also featured. Some studies involved a focus on early rehabilitation after surgery (ERAS) principles and how to optimise prehabilitation [5] and reduce length of hospital stay [6, 9, 10] readmission [11] and reoperation [12]. The effect of metal allergy [8] and drain usage [9] on outcomes is also explored. A variety of methodologies have been used including one randomised control trial [9], some machine learning [11] and three systematic reviews investigating the effect of metal allergy on outcome [8], associations with postoperative pain [10] and the effect of patella resurfacing [12].

## Summary of the included studies

The association of ipsilateral hip abductors muscle mass and gait following THA was assessed by Yasuda et al. [5] in a cohort of 42 patients. The cross-sectional area of gluteus maximus and the low-density lean tissue of gluteus medius and minimus were measured preoperatively and were found to be independently associated with gait speed and Timed Up-and-Go test 6-months following THA surgery, respectively. The authors concluded that preoperative optimisation and strengthening of the ipsilateral hip abductors prior to surgery may aid recovery of gait function after THA. This could be incorporated into pre-rehabilitation programmes to maintain physical strength while waiting for THA surgery whilst potentially also improving postoperative recovery.

March et al. [6] investigated the effect of patient 'resilience' on hospital length of stay in a prospective study of 75 patients undergoing KA. Resilience (the ability to recover from stress) is thought to be a major psychological factor in enabling ongoing functional independence in patients with chronic health conditions, however, no association was demonstrated between resilience before surgery and acute hospital length of stay after surgery. However, symptoms on the Depression, Anxiety and Stress Scale-21 were associated with longer acute hospital length of stay. Preoperative screening for these symptoms may help to identify patients at risk of longer length of stay and is an area requiring further research.

Clement et al. [7] performed a retrospective cohort study of 5857 patients waiting for KA to identify associations with negative Euro-Qol 5 dimension (EQ-5D) scores indication a health state worse than death (WTD). They identified that 771 (13.2%) patients were in a WTD health state prior to KA and that this was independently associated with increasing social deprivation and worse preoperative joint specific function (Oxford score). The Oxford knee score was found to be a reliable predictor of WTD state with a threshold value of 16 or less being 80% sensitive and specific. However, a WTD heath state was not associated with a worse improvement in joint specific function, health related quality of life, or patient satisfaction. The reason(s) why social deprivation was associated with a state WTD needs to be investigated further and addressed to ensure equity of care.

Peacock et al. [8] undertook a systematic review to assess the prevalence of Nickel hypersensitivity and its association with patient reported outcomes. They identified 20 studies including 1354 KAs. Nickel hypersensitivity prevalence varied widely from 0% to 87.5%. One study demonstrated an increased (4.2%) sensitivity following surgery, but three studies reported lower prevalence of hypersensitivity postoperatively. Evidence for the effect on patient reported outcomes was conflicting: seven studies reporting possible adverse clinical outcomes; six found no relationship. The authors assert that patch testing remains the prevailing approach for hypersensitivity diagnosis. After excluding common causes of implant failure, postoperative testing is advised for patients exhibiting suspected hypersensitivity and revision with hypoallergenic implants could be considered. This area continues to be a controversial and demands more research, to establish whether hypoallergenic implants, that are often two to three times more expensive, are justified and if so in which patients.

Maliarov et al. [9] conducted a randomised control trial to assess the effect of suction drainage following KA on early postoperative outcomes in addition to intravenous tranexamic acid. The study was powered to demonstrate a difference in haemoglobin level of 5 mg/L in the first 3 days. No difference was found between the groups on the third day and the no differences in length of stay, knee range of motion, of knee specific function. The use of suction drains after KA appears to be at the surgeon’s discretion.

Postoperative pain is estimated to effect approximately 23% of patients after THA. Zhang et al. [10] conducted a systematic review that included 54 studies to identify risk factors associated with pain after THA. They found an association between worse pain outcomes and female sex, poor preoperative pain or function, and more severe medical or psychiatric comorbidities. The association was found to be less strong between worse pain outcomes and preoperative high body mass index value, low radiographic grade arthritis, and low socioeconomic status. The authors conclude that modifiable factors should be optimised preoperatively and for those that are not modifiable patients at risk should be made aware of their potential outcome.

A risk prediction model for 30-day readmission after KA was developed by Gould et al. [11] using machine learning. They found that machine learning offered a slightly greater performance compared to traditional logistic regression models, but the discriminative performance of the prediction models remained poor. The advent of artificial intelligence in orthopaedics will likely result in a greater adoption of such technology that will aid decision making and predict outcomes to inform patients of their potential outcomes [13].

Shah et al. [12] under took a systematic review to examine whether patella resurfacing influences kneeling ability following KA. Of eight included studies (24,342 patients) two studies demonstrated an association between patella resurfacing and kneeling, but these were contrasting with one showing improved kneeling ability and the other the opposite. The rate of re-operation was significantly higher in those who had not undergone patella resurfacing. The question of whether the patella should be resurfaced or not continues. This should be reassessed in light of new technologies, such as robotic and kinematic alignment restoring the patellofemoral joint, and modern patella friendly knee arthroplasties [14].

## Conclusion

This special edition has advanced our knowledge of factors influencing to the outcome of hip and knee arthroplasty but has also identified several areas of research that need to be addressed to improve the outcomes of our patients.

## Data Availability

Not applicable.

## Abbreviations

EQ-5D: EuroQol 5-dimension
ERAS: Enhanced recovery after surgery
PROMs: Patient reported outcome measures
THA: Total hip arthroplasty
KA: Knee arthroplasty
WTD: Worse than death
